# Identification of Sex-Specific Markers Through 2b-RAD Sequencing in the Sea Urchin (*Mesocentrotus nudus*)

**DOI:** 10.3389/fgene.2021.717538

**Published:** 2021-08-05

**Authors:** Zhouping Cui, Jian Zhang, Zhihui Sun, Bingzheng Liu, Chong Zhao, Yaqing Chang

**Affiliations:** ^1^Key Laboratory of Mariculture and Stock Enhancement in North China Sea, Ministry of Agriculture and Rural Affairs, Dalian Ocean University, Dalian, China; ^2^School of Life Science, Liaoning Normal University, Dalian, China

**Keywords:** 2b-RAD, sex-specific marker, sea urchin, genome survey, SNP

## Abstract

Sex-specific markers play an important role in revealing sex-determination mechanism. Sea urchin (*Mesocentrotus nudus*) is an economically important mariculture species in several Asian countries and its gonads are the sole edible parts for people. However, growth rate and immunocompetence differ by sex in this species, sex-specific markers have not been identified, and the sex-determination mechanism of sea urchin remains undetermined. In this study, type IIB endonuclease restriction-site associated DNA sequencing (2b-RAD-seq) and a genome survey of *M. nudus* were performed, and three female-specific markers and three female heterogametic single nucleotide polymorphism (SNP) loci were identified. We validated these sex-specific markers via PCR amplification in a large number of individuals, including wild and artificially bred populations. Several open reading frames (ORFs) were predicted, although there are no potential genes known for sex determination and sex differentiation within the scaffold in which the sex-specific markers are located. Importantly, the female-specific sequences and female heterozygous SNP loci indicate that a female heterogametic and male homogametic ZW/ZZ sex-determination system should exist in *M. nudus*. The results provide a solid basis for revealing the sex-determination mechanism of this species, and open up new possibilities for developing sex-control breeding in sea urchin.

## Introduction

Sexual dimorphism is common in aquaculture animals, and growth rate, immunocompetence, and body size generally differ significantly by sex (Mei and Gui, 2015; Jiang et al., 2017). Sea urchin is widely distributed and commercially harvested all over the world (Inomata et al., 2016), and the gonads are the only edible parts for people. In addition, edible sea urchins are important mariculture species in several Asian countries. This group of animals shows gender-specific differences in immune response, for example, the immunocompetence of female *Paracentrotus lividus* urchins might be superior to that of males (Arizza et al., 2013). Moreover, the gonadal growth rate in *Abatus cavernosus* urchins is faster in males than in females (Gil et al., 2020), and the types and the contents of free amino acids, as well as lipid concentrations, are also closely linked to sexual dimorphism in urchins (Osako et al., 2007; Martínez-Pita et al., 2010; Díaz de Vivar et al., 2019). However, most sea urchins lack conspicuous sexually dimorphic phenotypes, making it impossible to distinguish the phenotypic sex of living animals. This dramatically increases the costs and reduces the efficiency of genetic breeding. In addition to commercial value, sea urchins are excellent organisms for the study of early embryonic development (Juliano et al., 2010), interactions among genes (Yuh et al., 1998; Oliveri et al., 2002), evolution (Kober and Bernardi, 2013), fertilization (Chun et al., 2014), and immunity (Branco et al., 2014). Therefore, it is important to reveal the genetic mechanism of sex determination in this species. To do so, it is critical to develop reliable genetic sex-identification markers (Kobayashi et al., 2013; Liu et al., 2013; Mei and Gui, 2015; Chen et al., 2018). A successful system would improve breeding efficiency and make it possible to implement sex-control breeding in these echinoderms (Wang et al., 2009; Martínez et al., 2014).

Several methods have been applied to identify sex-specific molecular markers, including traditional methods and high-throughput sequencing methods. Amplified fragment length polymorphism (AFLP) is a traditional method to score random SNP markers across an entire genome. However, this approach is expensive and requires complex experiments (Griffiths and Orr, 1999; Flachowsky et al., 2001). With the development of next-generation sequencing, several methods have been developed to obtain thousands of SNPs in a single experiment, such as double-digest restriction-site-associated DNA sequencing (dd-RAD-seq), sequence-based genotyping (SBG) and type IIB endonucleases restriction-site-associated DNA sequencing (2b-RAD-seq). Dd-RAD-seq technology can generate number of SNPs allowing for the construction of phylogenetic trees, detection of genetic variation of a population and accurate genetic maps (Zhou et al., 2014; Puchta et al., 2018). Due to the multiplexing a large number of samples, dd-RAD-seq requires the appropriate tools for analysis (Peterson et al., 2012). Using SBG can achieve genome-wide SNP discovery and genotyping of large populations without the information of a reference genome sequence, and this approach had been successfully used in the plants (Truong et al., 2012). Compared to the other traditional RAD-seq methods, 2b-RAD-seq is cost-effective and simple method, and allows for nearly every restriction site in the genome to be screened in parallel (Wang et al., 2012). Some studies have already successfully applied the 2b-RAD approach to explore sex-specific DNA fragments and SNPs in a great diversity of aquatic organisms (Liu et al., 2018; Zhou et al., 2019; Zhu et al., 2021). In addition to quickly identify genetic sex, sex-specific markers also provide insights into the sex-determination system in various animals (Liu et al., 2018). In several species of *Scylla* crab, sex-specific SNPs have been identified by 2b-RAD-seq, among which female are heterogamety and male are homogamety, while indicating that a ZW/ZZ sex-determination system may exist in the above organisms (Shi et al., 2018). In *Mastacembelus armatus*, two male-specific 2b-RAD tags and two SNPs were identified through 2b-RAD-seq, and reported that the *Tcl* gene was a candidate sex-determination gene (Xue et al., 2020). However, few studies have identified sex-specific markers in sea urchins.

*Mesocentrotus nudus* belongs to the Strongylocentrotidae family of Echinodermata, and is mainly distributed and farmed in coastal areas of several Asian countries, such as China, South Korea, and Japan (Agatsuma, 2020). To further understand the sex-determination mechanism in this species, we used 2b-RAD-seq to identify sex-specific markers. Subsequently, we verified the candidate markers in a large number of samples, including laboratory breeding population, cultured population, and wild populations, and perfect accordance was observed between sex-specific makers and sexual phenotype. Our results can be used to significantly improve breeding efficiency, and provide useful information for understanding the sex-determination mechanisms in sea urchins.

## Materials and Methods

### Sample Collection and Preparation

A total of 19 sea urchin individuals were collected from the Key Laboratory of Mariculture and Stock Enhancement in North China Sea, Ministry of Agriculture and Rural Affairs, Dalian Ocean University. In addition, aquaculture populations of *M. nudus* including 95 individuals were purchased from markets in Dalian Haibao Fishery Limited Company, China. Finally, 19 wild sea urchins were fished near Xixiakou, Shandong Province, China. Tube feet tissues from each individual were sampled and stored in absolute ethanol at −20°C for genomic DNA extraction. To determine the gender of each sea urchin, the corresponding gonad tissues were sampled in 4% paraformaldehyde (PFA) for histological examination.

### DNA Extraction

Genomic DNA was extracted following the protocols of a marine animal tissue genomic DNA extraction kit (Tiangen, DP324). The quality and concentration of DNA was determined by 1% agarose gel electrophoresis and using a SimpliNano spectrophotometer (Biochrom, United Kingdom).

### Gonadal Histology and Identifying of Gender

Samples of gonad tissues were fixed in PFA at 4°C, then washed three times with phosphate buffered solution (PBS) at room temperature. After incubating the samples in 30% sucrose for 3 h, they were embedded in optimal cutting temperature (OCT) compound and sectioned at 3 μm. Then they were stained with hematoxylin/eosin. The gender of each sea urchin was determined by observing the gonadal structure with a microscope (Leica DM4B microscope).

### Library Construction and 2b-RAD Sequencing

The genomic DNA of 20 individuals, including 10 males and 10 females, was used for 2b-RAD-seq. The 2b-RAD libraries were constructed at Qingdao OE Biotech Co., Ltd. (Qingdao, China) following previous methods with slight modification (Wang et al., 2012). Briefly, 200 ng genomic DNA from each individual was digested with 1 U *Bsa*XI restriction enzyme, then 10 μL digestion product was ligated with adaptor. Next, adaptor primers were used to amplify the ligation products by PCR. The quality and concentration of each PCR product was determined on an 8% polyacrylamide gel. Purified PCR products were digested with 2U *Sap*I restriction enzyme at 37°C for 30 min, and the digested products were further purified using streptavidin-coated magnetic beads. The supernatant was incubated with T4 DNA ligase at 16°C for 45 min, followed by purification of the ligation products. Barcodes were introduced by PCR with barcode-bearing primers, the PCR products were purified before sequencing. Finally, each library was pooled for sequencing using the Illumina Nova PE150PE platform.

### Data Filtering, Survey Genome, and Screening of Candidate Sex-Specific Markers

Paired-end reads were merged using Pear software (version 0.9.6) (Zhang et al., 2014), and terminal tag sequences were excluded from each output read. Reads with ambiguous bases (N) exceeding 8%, poor-quality reads, and those without restriction sites were excluded from the raw data. Ultimately, clean reads 33 bp in length were obtained. For the genome survey, DNA was extracted from one female and one male *M. nudus*. Libraries were constructed and sequenced on the Illumina NovaSeq platform (Illumina Inc., United States). Subsequently, 150 bp paired-end reads were clustered to build reference sequences for further locus genotyping using Ustacks software version 1.34 (Catchen et al., 2011). For genome survey, K-mer distribution was estimated by using jellyfish1 (version: 2.2.6)^1^ with parameters “-m 17 –C.” The genome size and heterozygosity ratio were estimated by the GenomeScope2. To identify candidate sex-specific markers, high-quality 33 bp reads were aligned against the genome survey using SOAP2 software (Li et al., 2009), and sequences with fewer than three reads were precluded (Li et al., 2009). Finally, candidate sex-specific markers for each individual were generated for further analyses.

### Validation of Sex Specific Sequences

According to the flank sequence of sex-specific markers, specific primers were designed using online software^2^ according to the flank sequences of the sex-specific marker. Then PCR reactions were carried out in a total volume of 20 μL, containing 10 μL 2 X Taq buffer (KangWei), 1 μL forward and reverse primer (10 mM), and 50–100 ng template DNA, adding sterile water to reach the final volume. The PCR amplification program was as follows: pre-denaturation at 94°C for 2 min, then 30 cycles of 94°C for 30 s, 58°C for 30 s, and a final extension of 2 min at 72°C. The PCR products were determined via 1% agarose gel electrophoresis and sequenced by Tsingke Biotechnology (Tianjin, China).

## Results

### 2b-RAD-Seq

In total, the 2b-RAD-seq produced 137,684,505 raw reads, 76,661,950 for females and 61,022,555 for males. After filtering low-quality reads, 72,125,643 clean reads for females and 56,991,085 clean reads for males were obtained. Based on the read quality and quantity, two female and two male samples were selected as references, clean reads were clustered and assembled. Subsequently, the reads of 20 individuals were aligned with reference samples, with an average alignment rate of 72.04%. After alignment analysis, 2,384,989 tags with an average depth of 36.78 × were obtained from the 20 individuals, and 43,922 polymorphic SNP markers were also screened (Table 1).

**TABLE 1:** Summary of 2b-RAD data obtained from 20 *M. nudus* individuals.

Name	Gender	Raw reads	Raw bases	Clean bases	Tags	Sequencing depth
F1	Female	7,416,917	7,211,370	6,997,835	119,303	39.37
F2	Female	7,416,917	7,211,370	6,977,732	131,127	36.83
F3	Female	7,416,917	7,211,370	6,964,296	115,465	40.94
F4	Female	7,416,917	7,211,370	6,962,948	116,298	40.65
F5	Female	7,416,917	7,211,370	6,973,281	121,724	37.85
F6	Female	7,915,473	7,696,999	7,465,187	119,963	41.41
F7	Female	7,915,473	7,696,999	7,475,314	131,311	38.88
F8	Female	7,915,473	7,696,999	7,432,458	116,989	42.4
F9	Female	7,915,473	7,696,999	7,440,918	118,280	42.67
F10	Female	7,915,473	7,696,999	7,435,674	123,705	40.27
M1	Male	5,672,850	5,486,113	5,343,733	116,336	31.43
M2	Male	5,672,850	5,486,113	5,341,739	126,894	29.32
M3	Male	5,672,850	5,486,113	5,318,442	111,114	33
M4	Male	5,672,850	5,486,113	5,307,239	111,615	33.18
M5	Male	5,672,850	5,486,113	5,282,468	115,196	30.92
M6	Male	6,531,661	6,290,517	6,072,984	118,336	34.98
M7	Male	6,531,661	6,290,517	6,095,777	127,515	33.51
M8	Male	6,531,661	6,290,517	6,105,512	111,501	37.54
M9	Male	6,531,661	6,290,517	6,077,488	112,770	36.65
M10	Male	6,531,661	6,290,517	6,045,703	119,547	34.15
Average	/	6,884,225.3	6,671,249.8	6,455,836.4	119,249.5	36.79
Total	/	137,684,505	133,424,995	129116728	2,384,989	/

### Genome Survey

Because the 2b-RAD tags were only 27 bp in length, too short to design PCR primers, and the genomic information for *M. nudus* is unavailable, a male and a female individual were subjected to short-read *de novo* sequencing. This generated 648,230,824 raw reads from the female; after filtering low-quality reads, 620,114,248 clean reads were acquired with a GC content of 37.06%. Additionally, a total of 669,375,890 raw reads were obtained from male individual, and 639,002,820 clean reads with a GC content of 37.23% were produced after filtering. The estimated genome size using SOAP *de novo* K-mer module (*K* = 17) was 626,279,328 bp with high heterozygosity (1.3%). The SRA raw reads were deposited in the GenBank public database with the accession number of PRJNA741812. Using these clean reads, a draft genome of each sex was assembled (1,582,413 scaffolds in female and 5,953,609 scaffolds in male). The longest scaffold was 52,656 bp with N50 lengths of 1,579 bp in the female genome, and 10,758 bp with N50 lengths of 150 bp in the male genome. Moreover, there were 78,550 (4.96%) and 943 (0.01%) scaffolds with lengths of more than 2 kb in the female and male draft genome, respectively (Table 2).

**TABLE 2:** Summery of genome survey dates.

	Female		Male	
	**Scaffold (length)**	**Scaffold (number)**	**Scaffold (lentgh)**	**Scaffold (lentgh)**
Number (>2,000 bp)	–	78,550	–	943
Max	52,656	–	10,758	–
N50	1,579	101,006	150	1,882,406
Total	817,576,203	1,582,413	1,076,599,617	5,953,609

### Identification of Candidate Sex-Specific Tags and SNPs

To identify putative sex-specific makers, the 2b-RAD tags and SNPs were aligned against the genome survey. After data filtering, we obtained 13 putative female-specific tags, which were present in 10 female samples (Table 3). In addition, 20,201 putative SNP loci were also obtained for further analysis. A smaller *p*-value of SNP means a higher correlation with sex; hence, we screened the top 10 sex-specific SNP locus with the lowest *p*-values for further analysis. Notably, the females appeared heterozygous whereas the males appeared homozygous for all 10-candidate sex-specific SNPs (Table 4), these data suggest that a ZW/ZZ sex-determination system exists in *M. nudus*.

**TABLE 3:** Information of candidate sex-specific tags.

Tag name	Ref ID	Sequence
Female-tag 1	ref7866-1	AACTCTGGAACCTTCCCTCCATCTTCC
Female-tag 2	ref62548-1	CCAAAAGCTACTTCATCTCCTGCAAGG
Female-tag 3	ref72559-1	CCTTACATTACGCTTTCTCCGACTGGC
Female-tag 4	ref75945-1	CGGCGGAAGACCGGAGCTCCATGTCAT
Female-tag 5	ref78854-1	CTAGATCTAACCTAGTCTCCTATGTTT
Female-tag 6	ref80771-1	CTCCACAGTACTTGTACTCCTTGACTC
Female-tag 7	ref102102-1	GCCTGGAGCACTGAATCTCCGTGCAAA
Female-tag 8	ref106304-1	GGACCTTTCACCTCTTCTCCTGTATGA
Female-tag 9	ref110721-1	GGTAGGAGCACTTTACCTCCCTCCTAA
Female-tag 10	ref129620-1	TATAGTTTTACCTTTTCTCCTTGTTCT
Female-tag 11	ref155669-1	TGTTTGCTCACTGGAGCTCCTTATGGA
Female-tag 12	ref159947-1	TTCAGAAAAACACTAGCTCCCTAAGAC
Female-tag 13	ref161941-1	TTCTAGAAAACTCTCACTCCACGAAAA

**TABLE 4:** Information of top 10 candidate sex-specific SNPs.

SNP name	Ref ID	SNP position	Reference base	Alternative base	Female	Male
1	ref32333	24	A	G	A/G	A/A
2	ref32333	26	T	A	T/A	T/T
3	ref68728	2	C	T	C/T	C/C
4	ref53064	3	C	T	C/T	C/C
5	ref107153	1	G	A	G/A	G/G
6	ref42855	9	A	C	C/C	A/A
7	ref125173	6	T	A	T/T	T/A
8	ref161496	8	G	T	G/G	G/T
9	ref86238	22	T	C	T/C	T/T
10	ref59409	3	T	C	T/C	T/T

### Validation of Candidate Sex-Specific Tags and SNPs

**FIGURE 1:**
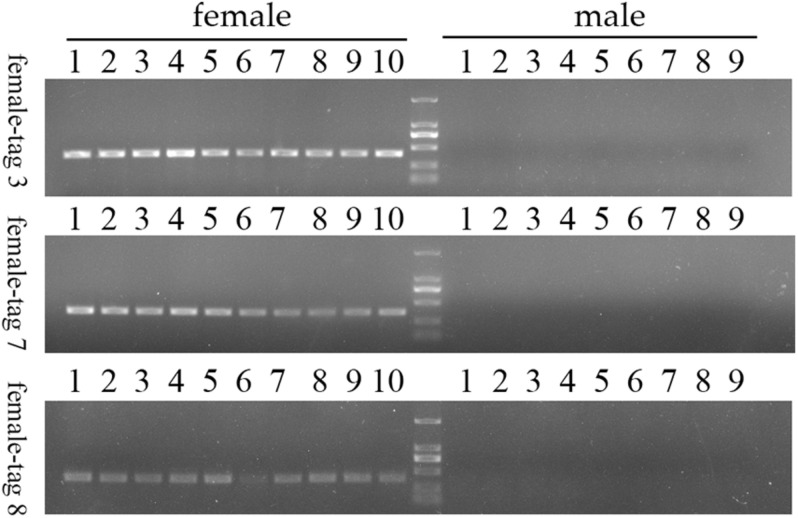
Validation of three candidate female-specific 2b-RAD-tags in 10 females and 9 males. Label M represents the DL2,000 DNA marker.

**FIGURE 2:**
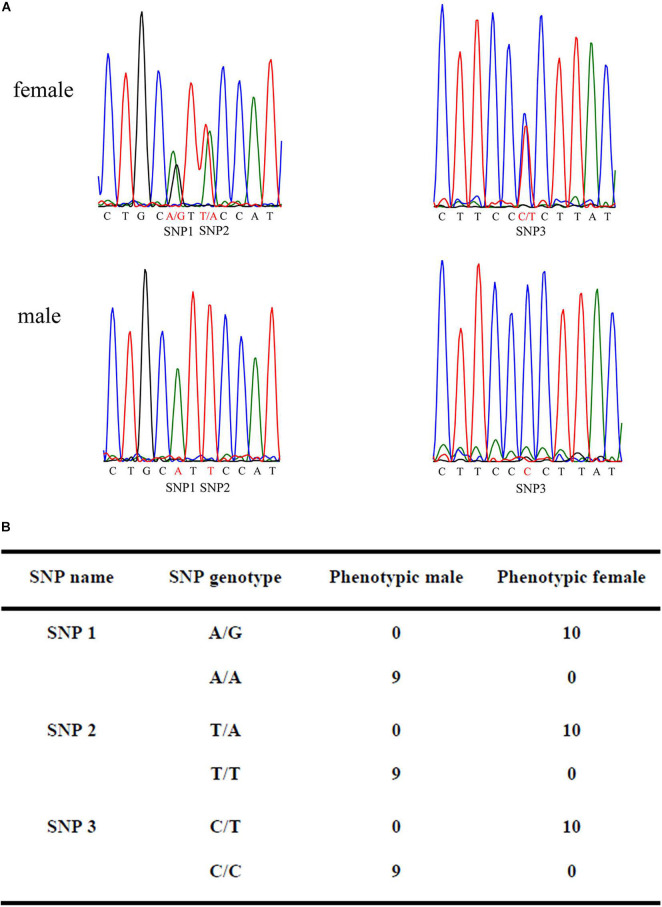
Validation of candidate sex-specific SNP loci in 10 females and 9 males. **(A)** Three female-specific SNP markers in the sequencing chromatograms of the sex-related sequence of *M. nudus*. **(B)** Statistics of sexual phenotype and SNP genotype of the 10 females and 9 males.

To confirm the authenticity of the candidate sex-specific tags and SNPs, primers were designed according to the flanking sequences of the 2b-RAD tags and SNPs from the genome survey (Table 5). Then, PCR reactions were performed using specific primers for 10 female and 9 male laboratory breeding sea urchins. As shown in Figure 1, the PCR products from 3 of the 13 female tags (female tags 3, 7, and 8) amplified one expected sizes band in females but not in males. Next, we purified these PCR products and sequenced them via Sanger sequencing. The nucleotide sequences were consistent with the female genomic sequences, further confirming the female-specific segments. Considering that the other 10 tags amplified products from both sexes (data not shown), these 10 tags were excluded from further study. In addition, the 10 candidate SNP loci were validated via PCR assays using specific primers from 20 individuals, and all PCR products were sequenced. Three of the candidate sex-specific SNPs were confirmed (Figure 2), and the others were excluded.

**TABLE 5:** primers used to verify candidate sex-specific tags and SNPs.

Sex-specific makers	Primer ID	Primer sequences (5′–3′)
ref72559-1	Female-tag 3-F Female-tag 3-R	GGAGTAGTGTCCCAATATCCG ACTACGACCCTGTGTTTGTTT
ref102102-1	Female-tag 7-F Female-tag 7-R	TGGCACTTTGGTGACAATACA ACAACCGCGAGTCTTGAAAT
ref106304-1	Female-tag 8-F Female-tag 8-R	ACTACCCTACCACAAAAGCA TTCATGCCTGATTCCGGTTG
ref32333	SNP1-F SNP1-R	CAAGGTGCTCTTGCGTGTA GGCTAAGCACGGTTATGAGA
ref32333	SNP2-F SNP2-R	CAAGGTGCTCTTGCGTGTA GGCTAAGCACGGTTATGAGA
ref68728	SNP3-F SNP3-R	TGAACAGAGCAATCCAGCCAT TTTGTGAAGCTGCGTGCAAAT

### Genetic Sex Identification in *M. nudus* Using Sex-Specific Tags

**FIGURE 3:**
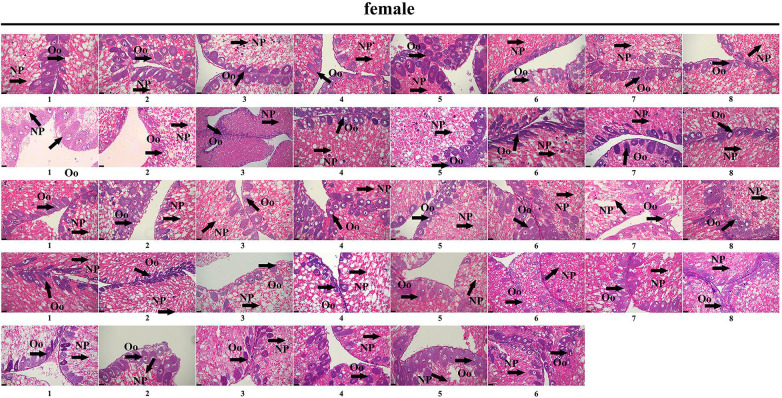
Identification of 38 females by histological detection of gonads in the breeding population. NP, nutritive phagocytes; Oo, oogonia.

**FIGURE 4:**
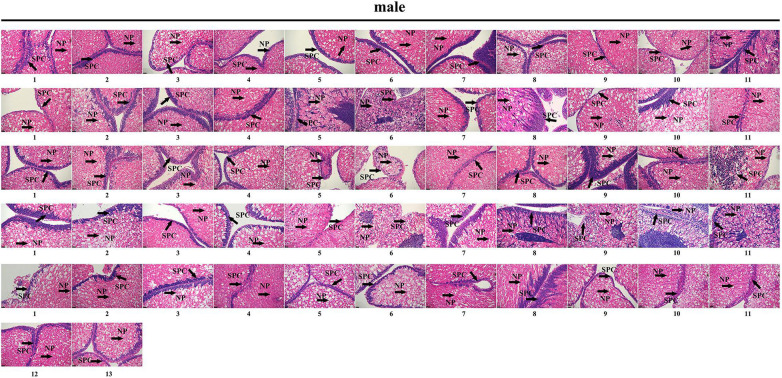
Identification of 57 males by histological detection of gonads in the breeding population. NP, nutritive phagocytes; SPC, spermatocyte.

**FIGURE 5:**
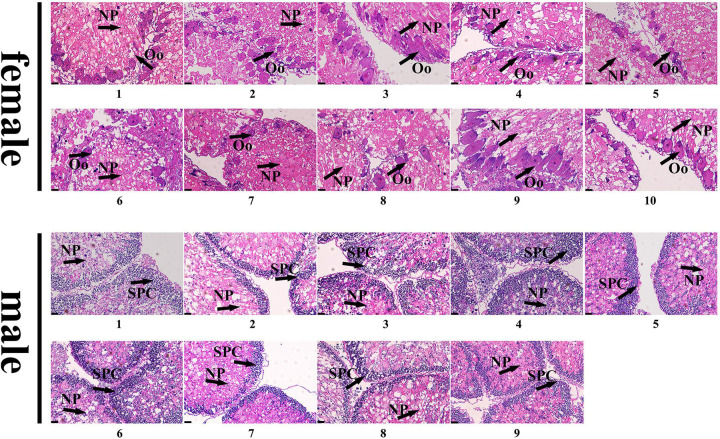
Identification of 10 females and 9 males by histological detection of gonads in the wild population. NP, nutritive phagocytes; Oo, oogonia; SPC, spermatocyte.

**FIGURE 6:**
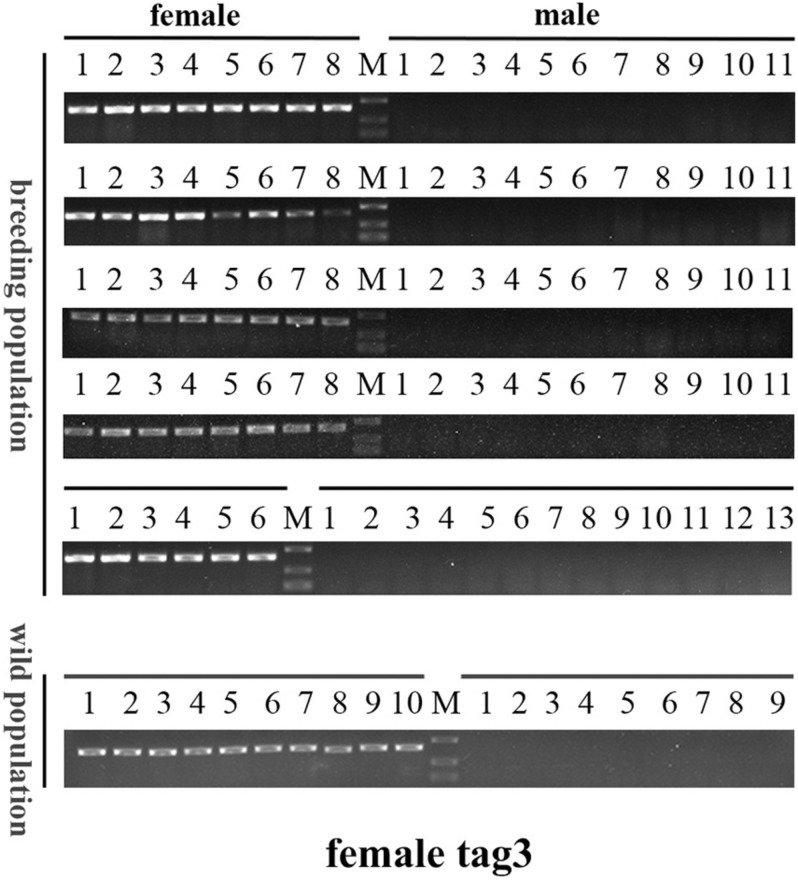
Electrophoretogram of the amplified products using female-tag 3 for genotypic sex identification in *M. nudus*. Label M represents the DL2,000 DNA marker (500 and 250 bp DNA ladder are shown).

**FIGURE 7:**
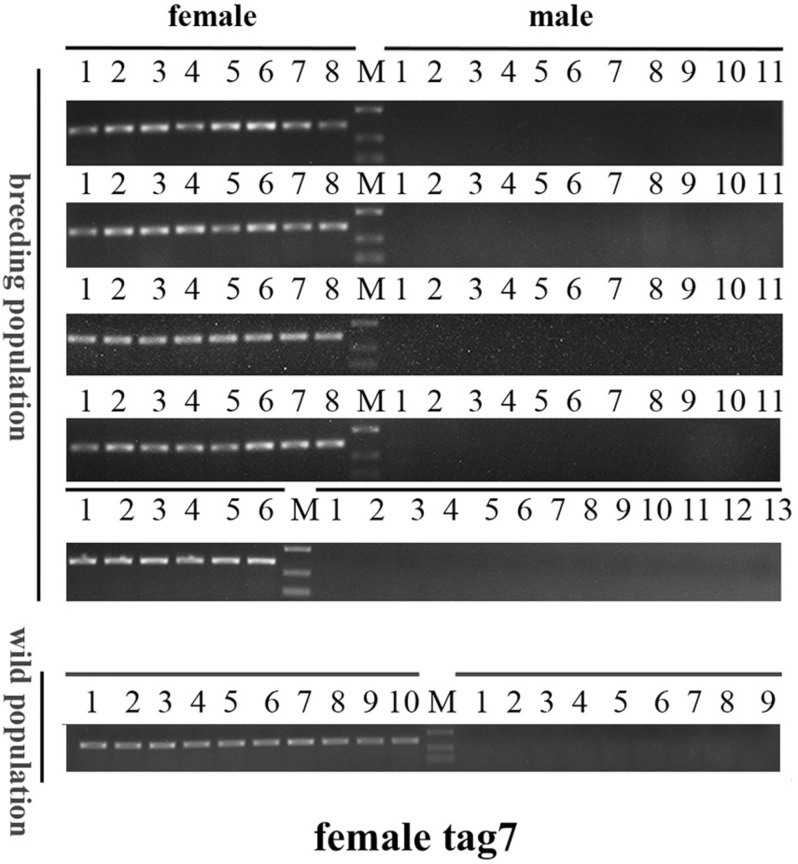
Electrophoretogram of the amplified products using female-tag 7 for genotypic sex identification in *M. nudus.* Label M represents the DL2,000 DNA marker (500 and 250 bp DNA ladder are shown).

**FIGURE 8:**
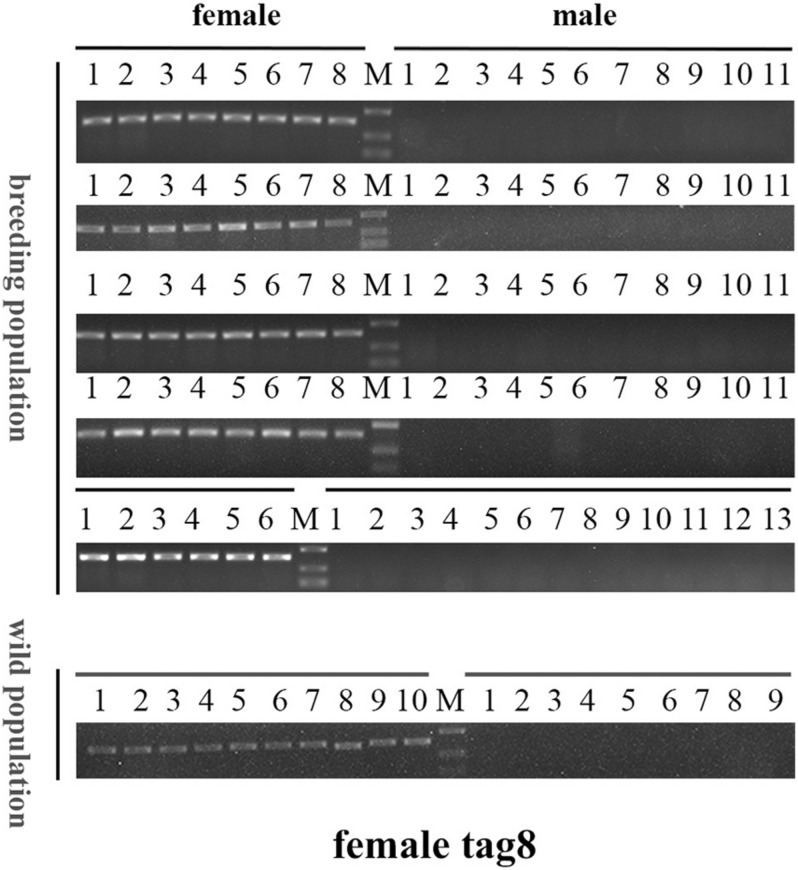
Electrophoretogram of the amplified products using female-tag 8 for genotypic sex identification in *M. nudus*. Label M represents the DL2,000 DNA marker (500 and 250 bp DNA ladder are shown).

To further validate the accuracy of the three female-specific tags, we performed PCR reactions on 95 sea urchins from breeding population outside the laboratory and wild population. Initially, the gender of each sea urchin was determined by gonadal histology, and 38 females (Figure 3) and 57 males (Figure 4) were identified in breeding population, 10 females and 9 males were identified in wild population (Figure 5). Then the three female-specific tags, including female-tag 3, female-tag 7, female-tag 8, were used to identify the genetic sex of these individuals. As expected, all three female-specific tags were only detected in the females, but not in males (Figures 6–8). These data suggest that the three female-specific markers could be used to identify the genetic sex of *M. nudus* with a very high rate of accuracy.

### Sex-Specific Sequence Annotation

**FIGURE 9:**
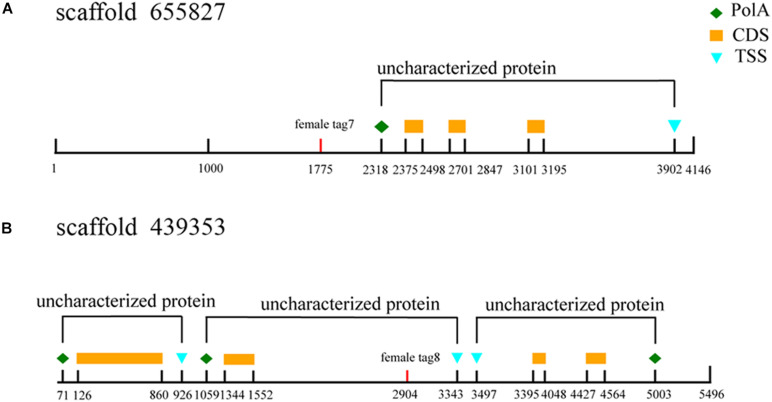
Annotation of sex-specific sequences obtained in *M. nudus*. Poly A, poly adenylic acid; CDS, coding sequence; TSS, transcriptional start site. **(A)** Annotation ofscaffold 655827. **(B)** Annotation of scaffold 439353.

The three female specific tags (female-tag 3, female-tag 7, female-tag 8) locate at scaffold 2838205, 655827, 439353, respectively. The scaffold 2838205 is 717 bp in length, and 655827, 439353 are 4,146 and 5,496 bp, respectively. To better understand these scaffolds, we predicated homologous genes by BLAST program. The databases of Softberry and NCBI, genomes of *Strongylocentrotus purpuratus* were used for annotation of the scaffolds. Although several open reading frames (ORFs) were predicted, there are no potential genes known for sex determination or differentiation within any scaffold (Figure 9). In addition, potential ORFs fail to be identified in the scaffold 2838205 due to a short sequence.

## Discussion

*Mesocentrotus nudus* is an economically important species in several Asian countries, but its benefits differ by sex and the gender of animals cannot be easily ascertained. In addition, sex-specific markers have not been identified, and the sex-determination mechanism of sea urchin remains undetermined. According to previous reports, the chromosomes of sea urchins are too small to be analyzed accurately, and heteromorphic sex chromosomes cannot be observed in most species (Saotome, 1987; Saotome et al., 2002; Eno et al., 2009). As an exception, a heteromorphic chromosome has been observed in *Paracentrotus lividus* sea urchins, for which an XX/XY sex-determination system has also been deduced (Lipani et al., 1996). In many animals, sex determination system has been deduced using sex-specific markers which developed by 2b-RAD-seq. In redtail catfish (*Mystus wyckioides*), some Y-specific fragments and X-specific fragments have been identified by 2b-RAD-seq, and XX/XY sex determination system was deduced because of the X-specific fragment showing dosage effect between females and males (Zhou et al., 2019). In this study, we revealed that all of the identified sex-specific markers were female-specific, and the identified SNP markers were heterozygous in females but homozygous in males (Figures 1, 2), our data suggest that a ZW/ZZ sex-determination system exists in *M. nudus*. However, it has been reported that the diploid chromosome number of *M. nudus* is 42 (2n = 42), and no heteromorphic sex chromosomes had not been observed (Saotome, 1987). In the future, we will use these female-specific sequences for chromosome fluorescence *in situ* hybridization (FISH) and attempt to locate sex chromosomes. Our results may help elucidate the origin of sex-determination systems.

Although it is difficult to observe heteromorphic sex chromosomes, sex-determining regions have been reported in several aquaculture animals (Wen et al., 2020; Xue et al., 2020). Liu et al. (2018) obtained a male-specific sequence 8,661 bp in length from both bighead carp (*Hypophthalmichthys nobilis*) and silver carp (*Hypophthalmichthys molitrix*) via 2b-RAD-seq, and suggested that it was located on the Y chromosome or in a nascent Y-linked region. Similarly, using male-specific 2b-RAD tags, Kirubakaran et al. (2019) identified a potential sex-determining region of 9 kb in Atlantic cod, and suggested that the *zkY* gene located in this region was a candidate sex-determining gene. In the present study, we identified three female-specific regions, providing a solid basis to reveal the sex-determination mechanisms in sea urchin. After homologous analysis by BLAST, several ORFs were predicted in these female-specific scaffolds, but no potential genes known for sex determination or differentiation within any scaffold. This phenome is similar to most previously reports that even though the sex-specific markers were extended into long segments within hundreds of genes, no sex related genes have been obtained (Pan et al., 2015; Shi et al., 2018; Zhou et al., 2019; Fang et al., 2020). In the future, we will use genome walking to analyze the differences in sequences between male and female individuals. The complete genome should also be sequenced to further elucidate the linkages among the three sex-specific sequences, as well as to reveal the sex-determination mechanism in sea urchin.

The sex-specific markers identified in our study were used to identify gender of more than one hundred of sea urchins with 100% accuracy (Figures 6–8). However, the need for DNA sample preparation limits the efficiency of this method. A direct PCR method would significantly reduce the identification time (Wang et al., 1993; Satya et al., 2013; Gao et al., 2020). This method uses NaOH-incubated products rather than purified DNA as template, and DNA can be extracted in 30 min from the tube feet of sea urchins. Furthermore, we compared the effectiveness of PCR using two different DNA extraction methods (using a DNA extraction kit and the alkaline extraction DNA procedure) and perfect consistency was observed between the two (data not shown). However, most aquaculture farms do not have PCR equipment and other required devices, which limits the application of this method. The loop-mediated isothermal amplification (LAMP) method can quickly amplify DNA with high specificity and efficiency under isothermal conditions, and LAMP equipment is not expensive (Notomi et al., 2000; Nagamine et al., 2002). Therefore, it would be of great value to develop a rapid sex-identification system that combines LAMP technology with female-specific markers.

## Conclusion

In conclusion, we identified sex-specific markers and SNPs via 2b-RAD-seq and a genome survey in *M. nudus*. These were found to belong to three female-specific scaffolds and were successfully used to accurately identify genetic sex of *M. nudus*. In addition, a ZW/ZZ sex-determination system was deduced for *M. nudus*, based on the presence of female-specific markers and the sex-specific SNPs in females appeared heterozygous but homozygous in males. Taken together, our results will help improve breeding efficiency, and provide a powerful tool for uncovering sex-determination mechanisms in sea urchins.

## Data Availability Statement

The datasets presented in this study can be found in online repositories. The names of the repository/repositories and accession number(s) can be found below: https://www.ncbi.nlm.nih.gov/genbank/, PRJNA741812.

## Ethics Statement

Ethical review and approval was not required for the animal study because sea urchin belongs to invertebrates, all animal experiments in this study are carried out with the permission of the local government.

## Author Contributions

ZS and YC: conceptualization and resources. ZC and JZ: methodology. ZC, JZ, and BL: investigation. ZC and CZ: data curation. ZS, ZC, and JZ: writing—original draft preparation. ZS: writing—review and editing, funding acquisition. All authors have read and agreed to the published version of the manuscript.

## Conflict of Interest

The authors declare that the research was conducted in the absence of any commercial or financial relationships that could be construed as a potential conflict of interest.

## Publisher’s Note

All claims expressed in this article are solely those of the authors and do not necessarily represent those of their affiliated organizations, or those of the publisher, the editors and the reviewers. Any product that may be evaluated in this article, or claim that may be made by its manufacturer, is not guaranteed or endorsed by the publisher.

## Footnotes

1
http://www.genome.umd.edu/jellyfish.html

2
http://biotools.nubic.northwestern.edu/OligoCalc.html
